# Prodromal and Early bvFTD: Evaluating Clinical Features and Current Biomarkers

**DOI:** 10.3389/fnins.2019.00658

**Published:** 2019-06-21

**Authors:** Kasper Katisko, Antti Cajanus, Titta Korhonen, Anne M. Remes, Annakaisa Haapasalo, Eino Solje

**Affiliations:** ^1^Institute of Clinical Medicine – Neurology, University of Eastern Finland, Kuopio, Finland; ^2^Neuro Center, Neurology, Kuopio University Hospital, Kuopio, Finland; ^3^Research Unit of Clinical Neuroscience, Neurology, University of Oulu, Oulu, Finland; ^4^Medical Research Center, Oulu University Hospital, Oulu, Finland; ^5^A.I. Virtanen Institute for Molecular Sciences, University of Eastern Finland, Kuopio, Finland

**Keywords:** frontotemporal dementia, frontotemporal lobar degeneration, diagnostics, differential diagnostics, biomarker, neuroimaging, GRN, *C9orf72*

## Abstract

Despite the current diagnostic criteria, early diagnostics of behavioral variant of frontotemporal dementia (bvFTD) has remained challenging. Patients with bvFTD often present with misleading psychiatric phenotype, and, on the other hand, impairment in memory functions have increasingly been reported. However, impaired episodic memory is currently considered as an exclusion criterion for bvFTD. Single biofluid-based or imaging biomarkers do not currently provide sufficient sensitivity or specificity for early bvFTD diagnosis at single-subject level, although studies have suggested improved accuracy with different biomarker combinations. In this mini review, we evaluate the core clinical features of early bvFTD and summarize the most potential imaging and fluid biomarkers for bvFTD diagnostics.

## Introduction

Frontotemporal lobar degeneration (FTLD) is the second most common early onset memory disorder (Ratnavalli et al., 2002), accounting for approximately 10 % of all progressive neurodegenerative memory diseases. FTLD has a substantial hereditary nature, and the most common genetic causes are the hexanucleotide repeat expansion in *C9orf72* gene (later *C9orf72-*RE) and mutations in *MAPT* or *GRN* genes. The most common clinical subtype of FTLD is the behavioral variant frontotemporal dementia (bvFTD), covering over half of the FTLD cases (Johnson et al., 2005). The diagnostics of bvFTD is challenging. In bvFTD, deterioration of episodic memory functions are not typically observed similarly to other neurodegenerative memory disorders and the patients are mainly characterized by altered personality and behavior (Rascovsky et al., 2011). Thus, the correct diagnosis is often delayed and misdiagnoses as psychiatric disorders are frequent (Galimberti et al., 2015; Solje et al., 2015).

The main inadequacy of the current diagnostic criteria is the lack of specificity. The *possible bvFTD* criteria include only behavioral symptoms and thus bvFTD can be easily confused with psychiatric disorders. The *probable bvFTD* criteria include also atrophy or hypoperfusion in frontal and/or temporal lobes and significant functional decline in addition to behavioral symptoms. Visual assessment of brain MRI results requires an experienced neuroradiologist and is time consuming, yet it provides only moderate sensitivity and specificity (Harper et al., 2016). Furthermore, as brain atrophy develops slowly, the observation of subtle brain changes in the early phase of the disease can be even more difficult.

Currently there are no routinely used and validated specific CSF or blood biomarkers for the diagnostics of bvFTD. However, there is an urgent need for such biomarkers for differential diagnostics, disease monitoring, and assessment of the effects of potential therapeutic treatments in FTLD patients. Biomarkers showing specific changes already at the presymptomatic or prodromal phase of the disease would be especially valuable for disease prediction and intervention when pharmacological, lifestyle, or psychosocial interventions become available. They would also be very useful for patient stratification in drug trials and would allow personalized medicine approaches for treatment and managing FTLD patients in the future.

In this minireview, we summarize core clinical features of early bvFTD and recent findings from studies examining novel brain imaging methods and biofluid biomarkers focusing on the early alterations in prodromal bvFTD. We attempt to provide some answers to the following questions. What are the most promising biomarkers for early bvFTD diagnostics? Is it possible to develop more sensitive multimodal diagnostic criteria or instruments to detect bvFTD during the early stages of the disease compared to the prevailing (Rascovsky et al., 2011) diagnostic criteria?

### Early Clinical Features in bvFTD Patients

The characteristic behavioral features in bvFTD patients may be measured with scales such as frontal behavioral inventory (FBI), neuropsychiatric inventory (NPI), Cambridge behavioral inventory (CBI) or other similar ratings that are preferably based on overt behaviors rather than inferences about the patient‘s cognitive state (Rascovsky et al., 2011). Presymptomatic familial FTLD patients with known mutations have been reported to present cognitive changes in neuropsychological testing up to eight years before the estimated onset of symptoms (Geschwind et al., 2001; Janssen et al., 2005; Rohrer et al., 2008, 2015; Dopper et al., 2013; Jiskoot et al., 2016, 2018b). Changes in attention, executive function, and social cognition have been found particularly in presymptomatic *MAPT* and *GRN* mutation carriers (Geschwind et al., 2001; Dopper et al., 2013; Rohrer et al., 2015; Jiskoot et al., 2016, 2018b). Neuropsychological tools assessing ventromedial prefrontal cortex dysfunction, such as theory of mind (ToM) tasks (Poletti et al., 2012; Pardini et al., 2013; Dodich et al., 2016) and social cognition and emotional assessment (SEA), have been demonstrated to be able to detect and distinguish bvFTD at the very early stage from Alzheimer’s disease (AD), and amnestic mild cognitive impairment (MCI) (Funkiewiez et al., 2012; Bertoux et al., 2013). Early decline in both affective and cognitive ToM component tasks has been noted in bvFTD patients, suggesting that impaired ventromedial prefrontal cortex function may explain the characteristic symptoms of bvFTD, such as early behavioral dysfunction, and loss of empathy (Gregory et al., 2002; Adenzato et al., 2010; Poletti et al., 2012).

Early neuropsychiatric symptoms and early contacts to psychiatric services are characteristic features in especially the inherited bvFTD associated with the *C9orf72-RE* (Boeve et al., 2012; Mahoney et al., 2012; Snowden et al., 2012; Devenney et al., 2014; Solje et al., 2015). In particular, delusions and hallucinations are prevalent (Omar et al., 2009; Boeve et al., 2012; Kertesz et al., 2013), and predominantly the patients with a concomitant motoneuron disease (MND) may experience psychotic symptoms in the prodromal stages of the disease (Velakoulis et al., 2009; Lillo et al., 2010). In a Finnish study, up to 60% of bvFTD patients carrying the *C9orf72-*RE presented with psychiatric symptoms on average 4.6 years prior to diagnosis of bvFTD (Solje et al., 2015). Also, criminal behavior has been identified in about 30% bvFTD (Diehl-Schmid et al., 2013; Liljegren et al., 2015; Shinagawa et al., 2017). According to these studies, the most common criminal acts include theft, traffic violations, trespassing, willful damage to property, housebreaking, assault, sexual advances, and indecent behavior.

Impaired episodic memory has been considered as an exclusion criterion for bvFTD in the current diagnostic criteria (Rascovsky et al., 2011), but increasing evidence has suggested impairment of memory functions and subjective memory complaints in early stages of bvFTD (Hodges et al., 2004; Pijnenburg et al., 2004; Hornberger et al., 2010; Hornberger and Piguet, 2012). In approximately 60% of cases, bvFTD patients and their caregivers report episodic memory disturbances in the initial stages of the disease (Hodges et al., 2004). Recent studies have also demonstrated that episodic memory impairments in bvFTD patients in neuropsychological tests may even be as severe as in patients with AD (Irish et al., 2011, 2014; Pennington et al., 2011; Bertoux et al., 2013; Frisch et al., 2013; Fernández-Matarrubia et al., 2017; Jiskoot et al., 2019).

### Early Imaging Findings in bvFTD Patients

The typical neuroimaging findings in bvFTD patients are atrophy and/or hypoperfusion of anterior temporal and frontal lobes, and these are included in the diagnostic criteria of probable bvFTD (Rascovsky et al., 2011). In the past 5–10 years, early structural and functional brain changes of bvFTD patients and presymptomatic carriers of the three main bvFTD-associated genetic mutations have been a target of intensive research. The focus of the studies has partially shifted from the conventional structural volumetric measures to measurements that are more novel, such as estimates of white matter tract integrity or functional network connectivity using diffusion weighted imaging and functional magnetic resonance imaging (MRI) methods and their quantification.

The earliest structural changes in presymptomatic mutation carriers can be detected in the insula and temporal and frontal areas as early as 10 years before the expected onset of the symptoms (Rohrer et al., 2015). Similar to symptomatic patients, the pattern of brain atrophy in the presymptomatic subjects varies depending on the mutation they carry (Lee et al., 2017; Bertrand et al., 2018; Cash et al., 2018; Olm et al., 2018; Wen et al., 2018). The characteristic brain changes according to predisposing genes are listed in Table 1. Presymptomatic *C9orf72-*RE carriers show earlier and more profound changes than *GRN* and *MAPT* carriers. However, similar findings are not evident in the offspring of MND patients carrying the *C9orf72-*RE (Walhout et al., 2015). In addition, a recent study with symptomatic bvFTD patients reported that increased cortical mean diffusivity is detected earlier than decreased cortical thickness, suggesting that diffusion-weighted imaging could be a preferable imaging modality also for detecting presymptomatic bvFTD patients (Illán-Gala et al., 2019).

**TABLE 1:** Summary of the early imaging findings of bvFTD according to different mutation carriers compared to non-carrying healthy controls.

**Group**	**Early imaging findings**	**References**
Presymptomatic C9ORF72-RE	Reduced volume in thalamus, cerebellum, parietal and frontal lobes. Reduced WM tracts connecting frontal lobes, thalamic radiation, corticospinal tracts.	Lee et al., 2017; Papma et al., 2017; Bertrand et al., 2018; Cash et al., 2018; Floeter et al., 2018; Jiskoot et al., 2018a; Popuri et al., 2018; Wen et al.,2018
Presymptomatic GRN	Reduced volume in insula, orbitofrontal, posterior frontal and anterior temporal lobes, and striatum. Reduced WM tracts in corpus callosum, superior longitudinal fasciculus and internal capsule.	Borroni et al., 2008; Pievani et al., 2014; Cash et al., 2018; Jiskoot et al., 2018a; Olm et al.,2018
Presymptomatic MAPT	Reduced volume in anterior and medial temporal lobes and the orbitofrontal lobe. Reduced WM tracts connecting frontal lobes.	Cash et al., 2018; Jiskoot et al.,2018a

According to the recent guidelines of European panel of experts (EANM-EAN), fluorodeoxyglucose positron emission tomography (FDG-PET) should be used in the early diagnosis of bvFTD, particularly because of its high negative predictive value (Grimmer et al., 2016; Arbizu et al., 2018; Caminiti et al., 2018). Characteristically bvFTD presents as uni- or bilateral hypometabolism in the prefrontal cortex, anterior temporal lobe, anterior cingulate, and basal ganglia (Morbelli et al., 2016; Krämer et al., 2018). However, the changes vary between individual patients, as some patients show a more prominent frontal hypometabolism, while the others present with a more prominent temporal lobe hypometabolism (Cerami et al., 2016). In patients with MCI, FDG-PET is useful in distinguishing patients with a progressive neurodegenerative disease from subjects with subjective symptoms or non-progressive conditions. However, at early stage, FDG-PET lacks specificity in differentiating between various neurodegenerative diseases (Arbizu et al., 2018).

The changes in spatially distinct, but functionally connected networks in cortical and subcortical areas in presymptomatic bvFTD patients have been evaluated using functional MRI (fMRI) methods. The main functional networks associated with bvFTD are the salience network (SN) and the default mode network (DMN) (Greicius et al., 2003; Menon and Uddin, 2010; Pievani et al., 2011; Farb et al., 2013; Lee et al., 2014). In symptomatic bvFTD patients, atrophy and hypoconnectivity in SN related areas is detected regardless of genetic etiology or neuropathologic presentation (Seeley et al., 2009; Zhou et al., 2010; Whitwell et al., 2011; Farb et al., 2013). Considering DMN, the studies show contradictory results, varying from hyper- to hypoconnectivity compared to healthy controls (Zhou et al., 2010; Whitwell et al., 2011; Farb et al., 2013; Rytty et al., 2013; Lee et al., 2014). In the presymptomatic *C9orf72-*RE carriers, there is hypoconnectivity in the SN compared to healthy controls, and this can be already distinguished in persons younger than 40 years of age (Lee et al., 2017).

Even though the results of recent neuroimaging studies appear promising in differentiating presymptomatic bvFTD from healthy controls, the imaging modalities and analysis methods need to be validated to obtain uniform protocols. In addition, also electro encephalogram and transcranial magnetic stimulation have been studied for diagnostic and even therapeutic purposes for bvFTD, but the results remain contradictory (Chan et al., 2004; Pijnenburg et al., 2008; Moretti et al., 2016; Carlino et al., 2014; Benussi et al., 2018). Moreover, large prospective studies are needed to validate the predictive value of different brain imaging techniques. Today, group level differences of presymptomatic bvFTD and healthy controls can be detected, but the predictive value of multimodal imaging at single-subject level can be considered suggestive at the most (Feis et al., 2018). Summary of the early imaging findings in bvFTD is provided in Table 1.

### Early Biological Fluid Biomarkers of bvFTD

The most studied biomarkers in the cerebrospinal fluid (CSF) of bvFTD patients have been phospho-tau, total tau, amyloid-β_1–42_ (and their ratios), and, more recently, neuronal cytoskeletal protein neurofilament light chain (NfL). Tau, phospho-tau and amyloid-β_1–42_ are validated biomarkers primarily in the diagnostics of AD. A few studies have shown that high CSF ratio of tau/amyloid-β_1–42_ or phospho-tau/amyloid-β_1–42_ can discriminate patients with AD from those with bvFTD (or FTLD in general) (Rivero-Santana et al., 2017; Paterson et al., 2018). However, decreased amyloid-β_1–42_ levels have been found in 25% of definite FTLD cases without any signs of AD (Kämälaïnen et al., 2015). Similarly to other neuronal cytoskeletal proteins like tau and phospho-tau, NfL release into the CSF is considered as a marker for neuronal injury and neurodegeneration. Altered levels of NfL can be detected in the CSF of patients with different brain diseases, suggesting that NfL may represent a general, rather unspecific marker for neurodegeneration. On the other hand, several studies have suggested that FTLD patients (including bvFTD) display higher levels of CSF NfL and/or lower ratio of phospho-tau/tau compared to healthy controls or patients with other neurodegenerative or psychiatric diseases, including e.g., AD (Skillbäck et al., 2014; Meeter et al., 2016; Vijverberg et al., 2017; Abu-Rumeileh et al., 2018; Goossens et al., 2018; Meeter L.H.H. et al., 2018; Niikado et al., 2018). Notably, combined assessment of NfL levels with the AD biomarkers in the CSF could provide additional value in the differentiation diagnostics of AD and bvFTD (de Jong et al., 2007). Additionally, concomitantly increased NfL levels and reduced phospho-tau/tau ratio in the CSF, and increased NfL levels in serum might show potential as biomarkers discriminating bvFTD from psychiatric disorders (Vijverberg et al., 2017; Al Shweiki et al., 2019).

It has been reported that elevated NfL levels and low phospho-tau/tau ratio in the CSF associate with poorer survival and especially with manifestation as motoneuron disease (FTLD-MND), suggesting that these biomarkers may be useful in disease monitoring in FTLD patients (Meeter et al., 2016; Ljubenkov et al., 2018; Meeter L.H.H. et al., 2018). In the same study, longitudinal data on CSF NfL levels did not indicate differences between control subjects and presymptomatic carriers of *GRN* or *MAPT* mutations or *C9orf72-*RE. However, the NfL levels showed a substantial increase after disease onset in all these patients, with the *GRN* mutation carriers showing the highest levels (Meeter et al., 2016). These data suggest that at individual level, NfL-test, with particular cut-off based reference values, may not necessarily be suitable in the very early stages of the disease when the neuropathological changes have already started to take place, but when there is not yet detectable neurodegeneration in the CNS. On the other hand, repeated analysis of CSF NfL levels at different time points during the very early stages of the disease in a patient could provide information about whether the NfL levels remain stable over time or show a rapid increase, allowing prediction of a potentially progressive neurodegenerative disease.

Reliable discrimination between FTLD-tau and FTLD-TDP, the two most common neuropathological subtypes of bvFTD patients (Perry et al., 2017), has remained challenging. Tau or TDP-43 levels in the CSF of neuropathologically confirmed FTLD-TDP and FTLD-tau cases were reported to show a great overlap (Kuiperij et al., 2017). Another study with neuropathologically confirmed FTLD-TDP and FTLD-tau patients suggested some other analytes, such as IL-17, IL-23, eotaxin-3 (CCL26), and macrophage-derived chemokine (MDC), as potential diagnostic biomarkers distinguishing between FTLD-TDP and FTLD-tau cases (Hu et al., 2010), and possibly reflecting involvement of different immunological processes in these neuropathological FTLD subtypes.

Investigations in FTLD patients with different genetic backgrounds have revealed that detection of CSF levels of dipeptide repeat proteins (DPR), namely poly-GP, in the *C9orf72-*RE carriers and progranulin in the *GRN* mutation carriers enables rather reliable separation of these patients from patients who do not carry these mutations (Ghidoni et al., 2012; Meeter et al., 2016; Gendron et al., 2017; Lehmer et al., 2017). On the other hand, poly-GP and progranulin levels in the CSF were increased already in the presymptomatic phase and did not show further significant increases during the symptomatic phase, thus suggesting that they do not predict disease onset or progression, but might be suitable for evaluating therapeutic effects (Meeter et al., 2016; Gendron et al., 2017; Lehmer et al., 2017). Furthermore, the poly-GP DPRs could be detected in the peripheral blood mononuclear cells (PBMCs) of the *C9orf72-*RE carriers, indicating that detection of poly-GP proteins in blood or other patient-derived cells might also be utilized in the diagnosis or therapy trials (Gendron et al., 2017). Also nuclear RNA foci, another pathological hallmark directly and specifically generated from the *C9orf72*-RE, can be detected for example in lymphocytes from the *C9orf72-*RE-carrying patients, and thus could potentially be exploited as biomarkers in assessing the effects of potential therapeutic substances, e.g., antisense oligonucleotides or small molecules (Su et al., 2014). However, it is still unclear at which point during the disease course these pathological hallmarks become detectable in different types of cells, and thus further studies are needed to evaluate their potential as early or prognostic peripheral biomarkers in *C9orf72-*RE carriers. Recent investigations have also suggested that decreased levels of *GRN* mRNA and progranulin in the serum can be used to identify affected and at-risk presymptomatic *GRN* mutation carriers from controls, suggesting potential as peripherally measurable biomarkers for these patients (Guven et al., 2019). Future longitudinal data on serum *GRN* mRNA or progranulin levels at different time points during the disease course would provide insights into their potential use as prognostic biomarkers.

Increasing amount of recent reports indicate a potential association between FTLD and inflammation. Therefore, assessing different inflammatory biomarkers in FTLD could provide diagnostic and/or prognostic value. Autoantibodies against AMPA receptor and antinuclear autoantibodies (ANA) were detected significantly more often in the sera of sporadic FTLD patients compared to control subjects (Borroni et al., 2017; Cavazzana et al., 2018). Levels of glial cell-derived inflammatory mediator YKL-40 (Chitinase 3-like 1) have been shown to be elevated in the CSF of both FTLD and AD patients compared to controls (Janelidze et al., 2016; Alcolea et al., 2017). On the other hand, similarly to several other biomarkers, also YKL-40 levels provide poor specificity between different neurodegenerative diseases (Alcolea et al., 2014; Janelidze et al., 2016). However, YKL-40 was found useful in the differential diagnostics between FTLD and psychiatric diseases, as higher YKL-40 levels in combination with higher NfL levels and reduced p-tau/tau ratio in the CSF could be used to distinguish FTLD patients from those with a psychiatric disease (Vijverberg et al., 2017). A key question considering the early diagnostics of FTLD is at which point during the disease course the inflammation occurs and can be detected. One of the important proteins related to inflammation is the triggering receptor expressed in myeloid cells 2 (TREM2), expressed in microglia. So far, only *GRN* mutations have been associated with elevated CSF soluble TREM2 (sTREM2) levels in FTLD (Woollacott et al., 2018). These data altogether thus suggest that specific inflammatory markers or a specific combination of them could have potential as early biomarkers of neurodegeneration and perhaps specifically also FTLD. The current CSF- or blood-derived biomarkers and their feasibility in the diagnostics of bvFTD are summarized in Table 2.

**TABLE 2:** Current CSF- or blood-derived biomarkers and their feasibility in the diagnostics of bvFTD.

**Biomarker**	**In clinical use (currently; yes/no)**	**Potential utility value**	**References**
CSF amyloid-β1–42	Yes; AD diagnostics	Useful in differentiating AD vs. bvFTD (decreased in AD).	Rivero-Santana et al., 2017; Paterson et al.,2018
CSF tau	Yes; AD diagnostics	Combined with decreased amyloid-β1–42 (tau/amyloid-β1–42 ratio), high levels indicate AD over other neurodegenerative diseases, including bvFTD.	Rivero-Santana et al., 2017; Paterson et al.,2018
CSF phospho-tau	Yes; AD diagnostics	Combined with decreased amyloid-β1–42, high levels indicate AD over other neurodegenerative diseases.	Rivero-Santana et al.,2017
CSF phospho-tau/tau ratio	No; Not routinely used	Especially low values observed in Creutzfeldt-Jakob disease. In general, lower values indicate more severe neurodegeneration (for example ALS or rapidly progressive FTLD), and indicate FTLD over psychiatric disorders or AD.	Riemenschneider et al., 2003; Pijnenburg et al., 2015; Vijverberg et al.,2017
CSF and blood NfL	No	Disease severity assessment and diagnostics between bvFTD and non-neurodegenerative diseases (psychiatric). Higher levels in bvFTD compared to AD have been observed. Similar results found in both blood and CSF.	Vijverberg et al., 2017; Steinacker et al., 2018; Abu-Rumeileh et al., 2018; Al Shweiki et al.,2019
Specific markers for genetic forms of FTLD	No	Detectable in presymptomatic phase. Potentially useful in the future when evaluating the effects of therapeutic interventions in genetic bvFTD.	Ghidoni et al., 2012; Su et al., 2014; Gendron et al.,2017
- CSF/blood poly-GP			
- Nuclear RNA foci from C9orf72-RE			
- CSF/blood progranulin			
Inflammatory markers	Yes; ANA detection is used in diagnostics of several systemic and especially rheumatic autoimmune conditions	Diagnostics between FTLD and non-neurodegenerative diseases. Might indicate inflammation as a potential target for therapeutic approach.	Vijverberg et al., 2017; Borroni et al., 2017; Cavazzana et al., 2018; Woollacott et al.,2018
- anti-AMPA GluA3			
- ANA			
- YKL-40			
s - sTREM2			

## Discussion

Despite recent advances in the early characterization of bvFTD, early clinical diagnosis is still a challenge. The primary symptoms of bvFTD include apathy, changes in personality, executive function deficits, and abnormal social behavior. According to current criteria, memory performance is not impaired at the early stage of the bvFTD, whereas memory loss and visuospatial problems are often the early symptoms in patients with AD (Dubois et al., 2007; Rascovsky et al., 2011; Ranasinghe et al., 2016). In contrast, patients with bvFTD may present with early neuropsychiatric symptoms, such as depression and psychosis, that are compatible with a range of neurologic and psychiatric disorders. Those patients, who lack the key symptoms for the clinical diagnosis of bvFTD and do not meet full diagnostic criteria, are initially often misdiagnosed as psychiatric disorders, and other neurological diseases, most often AD. Therefore, misrecognition of symptoms in the early stages of bvFTD frequently delays a correct diagnosis (Mendez and Perryman, 2002; Rosness et al., 2008; Landqvist Waldö et al., 2015; Bertoux et al., 2016). Despite the significant clinical overlap between bvFTD, other neurodegenerative diseases and psychiatric disorders, the clinical phenotype has a great emphasis in the current diagnostic criteria as the clinical phenotype solely forms the first level (possible bvFTD) of the diagnosis. Additionally, the further criteria require positive imaging findings (for probable bvFTD) and genetic confirmation (for definite bvFTD), although the imaging findings may be absent especially in the early stages of bvFTD. Moreover, most of the cases are sporadic without a known genetic alteration underlying the disease. Thus, diagnosing bvFTD solely based on the “possible bvFTD” criteria lacks specificity, but on the other hand it is often not possible to increase the diagnostic certainty to the “probable” or “definite” levels.

Due to the limitations in the current criteria, more sensitive and specific neuroimaging and biomarker assessments are needed for early and more accurate diagnosis. On the other hand, the current criteria should remain as the clinical gold standard at least until bvFTD-specific biological markers become available. Future biomarkers should ideally be able to differentiate FTLD patients with different underlying pathological processes or genetic backgrounds, as potential treatment strategies will likely be targeted for specific patients or group of patients in a more personalized manner. Additionally, identifying individuals at increased risk for FTLD before disease onset and at still early stages of neurodegeneration could enable earlier diagnostics and increase potential for interventions.

To date, individual imaging or biomarker analysis tools do not yet provide sufficient sensitivity and/or specificity. On the other hand, combining different diagnostic instruments (neuroimaging, serum NfL measurement, immunological/metabolic biomarkers, and neuropathological biomarkers) could result in greater applicability to clinical diagnostics, compared to the prevailing diagnostic criteria. For instance, combination of serum NfL and diffusion weighted MRI scans could provide greater sensitivity and specificity in differentiating bvFTD from other neurodegenerative and psychiatric disorders and could possibly be included in the diagnostic criteria in the future. However, the biomarkers considered in this review still require extensive research as the current literature for them in FTLD or bvFTD rests on only a few suggestive findings. As bvFTD can be considered a clinically, genetically and pathologically heterogenous disease, the composition of large and well-defined cohorts is necessary. This emphasizes the need of multicenter, international collaboration, as larger study populations are needed to validate and screen the existing and upcoming diagnostic tools for clinical use.

## Author Contributions

ES, AR, and AH contributed to the conception and design of the study. All authors wrote the sections, edited, read, and approved the final version of the manuscript for submission.

## Conflict of Interest Statement

The authors declare that the research was conducted in the absence of any commercial or financial relationships that could be construed as a potential conflict of interest.

## References

[B1] Abu-Rumeileh S. Mometto N. Bartoletti-Stella A. Polischi B. Oppi F. Poda R. (2018). Cerebrospinal fluid biomarkers in patients with frontotemporal dementia spectrum: a single-center study. *J. Alzheimers Dis.* 66 551–563. 10.3233/jad-180409 30320576

[B2] Adenzato M. Cavallo M. Enrici I. (2010). Theory of mind ability in the behavioural variant of frontotemporal dementia: an analysis of the neural, cognitive, and social levels. *Neuropsychologia* 48 2–12. 10.1016/j.neuropsychologia.2009.08.001 19666039

[B3] Al Shweiki M. Steinacker P. Oeckl P. Hengerer P. Danek A. Fassbender K. (2019). Neurofilament light chain as a blood biomarker to differentiate psychiatric disorders from behavioural variant frontotemporal dementia. *J. Psychiatr. Res.* 113 137–140. 10.1016/j.jpsychires.2019.03.019 30953863

[B4] Alcolea D. Carmona-Iragui M. Suárez-Calvet M. Sánchez-Saudinós M. B. Sala I. Antón-Aguirre S. (2014). Relationship between β-secretase, inflammation and core cerebrospinal fluid biomarkers for alzheimer’s disease. *J. Alzheimer’s Dis.* 42 157–167. 10.3233/JAD-140240 24820015

[B5] Alcolea D. Vilaplana E. Suárez-Calvet M. Illán-Gala I. Blesa R. Clarimón J. (2017). CSF sAPPβ, YKL-40, and neurofilament light in frontotemporal lobar degeneration. *Neurology* 89 178–188. 10.1212/WNL.0000000000004088 28592456

[B6] Arbizu J. Festari C. Altomare D. Walker Z. Bouwman F. Rivolta J. (2018). Clinical utility of FDG-PET for the clinical diagnosis in MCI. *Eur. J. Nucl. Med. Mol. Imaging* 45 1497–1508. 10.1007/s00259-018-4039-7 29704037

[B7] Benussi A. Alberici A. Ferrari C. Cantoni V. Dell’Era V. Turrone R. (2018). The impact of transcranial magnetic stimulation on diagnostic confidence in patients with Alzheimer disease. *Alzheimers Res. Ther.* 10:94. 10.1186/s13195-018-0423-6 30227895 PMC6145195

[B8] Bertoux M. de Souza L. C. O’Callaghan C. Greve A. Sarazin M. Dubois B. (2016). Social cognition deficits: the key to discriminate behavioral variant frontotemporal dementia from alzheimer’s disease regardless of amnesia? *J. Alzheimers Dis.* 49 1065–1074. 10.3233/JAD-150686 26756325

[B9] Bertoux M. Funkiewiez A. O’Callaghan C. Dubois B. Hornberger M. (2013). Sensitivity and specificity of ventromedial prefrontal cortex tests in behavioral variant frontotemporal dementia. *Alzheimers Dement.* 9 S84–S94. 10.1016/j.jalz.2012.09.010 23218606

[B10] Bertrand A. Wen J. Rinaldi D. Houot M. Sayah S. Camuzat A. (2018). Early cognitive, structural, and microstructural changes in presymptomatic C9orf72 carriers younger than 40 years. *JAMA Neurol.* 75 236–245. 10.1001/jamaneurol.2017.4266 29197216 PMC5838615

[B11] Boeve B. F. Boylan K. B. Graff-Radford N. R. DeJesus-Hernandez M. Knopman D. S. Pedraza O. (2012). Characterization of frontotemporal dementia and/or amyotrophic lateral sclerosis associated with the GGGGCC repeat expansion in C9ORF72. *Brain* 135 765–783. 10.1093/brain/aws004 22366793 PMC3286335

[B12] Borroni B. Alberici A. Premi E. Archetti S. Garibotto V. Agosti C. (2008). Brain magnetic resonance imaging structural changes in a pedigree of asymptomatic progranulin mutation carriers. *Rejuven. Res.* 11 585–595. 10.1089/rej.2007.0623 18593276

[B13] Borroni B. Stanic J. Verpelli C. Mellone M. Bonomi E. Alberici A. (2017). Anti-AMPA GluA3 antibodies in frontotemporal dementia: a new molecular target. *Sci. Rep.* 7:6723. 10.1038/s41598-017-06117-y 28751743 PMC5532270

[B14] Caminiti S. P. Ballarini T. Sala A. Cerami C. Presotto L. Santangelo R. (2018). FDG-PET and CSF biomarker accuracy in prediction of conversion to different dementias in a large multicentre MCI cohort. *NeuroImage. Clin.* 18 167–177. 10.1016/j.nicl.2018.01.019 29387532 PMC5790816

[B15] Carlino E. Frisaldi E. Rainero I. Asteggiano G. Cappa G. Tarenzi L. (2014). Nonlinear analysis of electroencephalogram in frontotemporal lobar degeneration. *Neuroreport* 25 496–500. 10.1097/WNR.0000000000000123 24717666

[B16] Cash D. M. Bocchetta M. Thomas D. L. Dick K. M. van Swieten J. C. Borroni B. (2018). Patterns of gray matter atrophy in genetic frontotemporal dementia: results from the GENFI study. *Neurobiol. Aging* 62 191–196. 10.1016/j.neurobiolaging.2017.10.008 29172163 PMC5759893

[B17] Cavazzana I. Alberici A. Bonomi E. Ottaviani R. Kumar R. Archetti S. (2018). Antinuclear antibodies in frontotemporal dementia: the tip’s of autoimmunity iceberg? *J. Neuroimmunol.* 325 61–63. 10.1016/j.jneuroim.2018.10.006 30391902

[B18] Cerami C. Dodich A. Lettieri G. Iannaccone S. Magnani G. Marcone A. (2016). Different FDG-PET metabolic patterns at single-subject level in the behavioral variant of fronto-temporal dementia. *Cortex* 83 101–112. 10.1016/j.cortex.2016.07.008 27498041

[B19] Chan D. Walters R. J. Sampson E. L. Schott J. M. Smith S. J. Rossor M. N. (2004). EEG abnormalities in frontotemporal lobar degeneration. *Neurology* 62 1628–1630. 10.1212/01.wnl.0000123103.89419.b7 15136699

[B20] de Jong D. Jansen R. W. Pijnenburg Y. A. L. van Geel W. J. A. Borm G. F. Kremer H. P. H. (2007). CSF neurofilament proteins in the differential diagnosis of dementia. *J. Neurol. Neurosurg. Psychiatry* 78 936–938. 10.1136/jnnp.2006.107326 17314187 PMC2117885

[B21] Devenney E. Hornberger M. Irish M. Mioshi E. Burrell J. Tan R. (2014). Frontotemporal dementia associated with the *C9ORF72* Mutation. *JAMA Neurol.* 71:331. 10.1001/jamaneurol.2013.6002 24445580

[B22] Diehl-Schmid J. Perneczky R. Koch J. Nedopil N. Kurz A. (2013). Guilty by suspicion? criminal behavior in frontotemporal lobar degeneration. *Cogn. Behav. Neurol.* 26 73–77. 10.1097/WNN.0b013e31829cff11 23812170

[B23] Dodich A. Cerami C. Crespi C. Canessa N. Lettieri G. Iannaccone S. (2016). Differential impairment of cognitive and affective mentalizing abilities in neurodegenerative dementias: evidence from behavioral variant of frontotemporal dementia, alzheimer’s disease, and mild cognitive impairment. *J. Alzheimers Dis.* 50 1011–1022. 10.3233/JAD-150605 26836153

[B24] Dopper E. G. Rombouts S. A. Jiskoot L. C. den Heijer T. de Graaf J. R. de Koning I. (2013). Structural and functional brain connectivity in presymptomatic familial frontotemporal dementia. *Neurology* 80 814–823. 10.1212/WNL.0b013e31828407bc 23390180 PMC3598452

[B25] Dubois B. Feldman H. H. Jacova C. Dekosky S. T. Barberger-Gateau P. Cummings J. (2007). Research criteria for the diagnosis of Alzheimer’s disease: revising the NINCDS-ADRDA criteria. *Lancet Neurol.* 6 734–746. 10.1016/S1474-4422(07)70178-3 17616482

[B26] Farb N. A. S. Grady C. L. Strother S. Tang-Wai D. F. Masellis M. Black S. (2013). Abnormal network connectivity in frontotemporal dementia: evidence for prefrontal isolation. *Cortex* 49 1856–1873. 10.1016/j.cortex.2012.09.008 23092697

[B27] Feis R. A. Bouts M. J. R. J. Panman J. L. Jiskoot L. C. Dopper E. G. P. Schouten T. M. (2018). Single-subject classification of presymptomatic frontotemporal dementia mutation carriers using multimodal MRI. *NeuroImage Clin.* 20 188–196. 10.1016/j.nicl.2018.07.014 30094168 PMC6072645

[B28] Fernández-Matarrubia M. Matías-Guiu J. A. Cabrera-Martín M. N. Moreno-Ramos T. Valles-Salgado M. Carreras J. L. (2017). Episodic memory dysfunction in behavioral variant frontotemporal dementia: a clinical and FDG-PET study. *J. Alzheimers Dis.* 57 1251–1264. 10.3233/JAD-160874 28304289

[B29] Floeter M. K. Danielian L. E. Braun L. E. Wu T. (2018). Longitudinal diffusion imaging across the C9orf72 clinical spectrum. *J. Neurol. Neurosurg. Psychiatry* 89 53–60. 10.1136/jnnp-2017-316799 29054917 PMC6454927

[B30] Frisch S. Dukart J. Vogt B. Horstmann A. Becker G. Villringer A. (2013). Dissociating memory networks in early Alzheimer’s disease and frontotemporal lobar degeneration - a combined study of hypometabolism and atrophy. *PLoS One* 8:e55251 10.1371/journal.pone.0055251 PMC3573064 23457466

[B31] Funkiewiez A. Bertoux M. de Souza L. C. Lévy R. Dubois B. (2012). The SEA (social cognition and emotional assessment): a clinical neuropsychological tool for early diagnosis of frontal variant of frontotemporal lobar degeneration. *Neuropsychology* 26 81–90. 10.1037/a0025318 21895376

[B32] Galimberti D. Dell’Osso B. Altamura A. C. Scarpini E. (2015). Psychiatric symptoms in frontotemporal dementia: epidemiology, phenotypes, and differential diagnosis. *Biol. Psychiatry* 78 684–692. 10.1016/j.biopsych.2015.03.028 25958088

[B33] Gendron T. F. Chew J. Stankowski J. N. Hayes L. R. Zhang Y. J. Prudencio M. (2017). Poly(GP) proteins are a useful pharmacodynamic marker for C9ORF72-associated amyotrophic lateral sclerosis. *Sci. Transl. Med.* 9:eaai7866. 10.1126/scitranslmed.aai7866 28356511 PMC5576451

[B34] Geschwind D. H. Robidoux J. Alarcón M. Miller B. L. Wilhelmsen K. C. Cummings J. L. (2001). Dementia and neurodevelopmental predisposition: cognitive dysfunction in presymptomatic subjects precedes dementia by decades in frontotemporal dementia. *Ann. Neurol.* 50 741–746. 10.1002/ana.10024 11761471

[B35] Ghidoni R. Stoppani E. Rossi G. Piccoli E. Albertini V. Paterlini A. (2012). Optimal plasma progranulin cutoff value for predicting null progranulin mutations in neurodegenerative diseases: a multicenter italian study. *Neurodegener. Dis.* 9 121–127. 10.1159/000333132 22123177

[B36] Goossens J. Bjerke M. Mossevelde S. Van den Bossche T. Goeman J. De Vil B. (2018). Diagnostic value of cerebrospinal fluid tau, neurofilament, and progranulin in definite frontotemporal lobar degeneration. *Alzheimers Res. Ther.* 10:31. 10.1186/s13195-018-0364-0 29559004 PMC5859717

[B37] Gregory C. Lough S. Stone V. Erzinclioglu S. Martin L. Baron-Cohen S. (2002). Theory of mind in patients with frontal variant frontotemporal dementia and alzheimer’s disease: theoretical and practical implications. *Brain* 125 752–764. 10.1093/brain/awf079 11912109

[B38] Greicius M. D. Krasnow B. Reiss A. L. Menon V. (2003). Functional connectivity in the resting brain: A network analysis of the default mode hypothesis. *Proc. Natl. Acad. Sci. U.S.A.* 100 253–258. 10.1073/pnas.0135058100 12506194 PMC140943

[B39] Grimmer T. Wutz C. Alexopoulos P. Drzezga A. Förster S. Förstl H. (2016). Visual versus fully automated analyses of 18F-FDG and amyloid PET for prediction of dementia due to alzheimer disease in mild cognitive impairment. *J. Nucl. Med.* 57 204–207. 10.2967/jnumed.115.163717 26585056

[B40] Guven G. Bilgic B. Tufekcioglu Z. Erginel Unaltuna N. Hanagasi H. Gurvit H. (2019). Peripheral GRN mRNA and serum progranulin levels as a potential indicator for both the presence of splice site mutations and individuals at risk for frontotemporal dementia. *J. Alzheimer’s Dis.* 67 159–167. 10.3233/JAD-180599 30475763 PMC6422018

[B41] Harper L. Fumagalli G. G. Barkhof F. Scheltens P. O’Brien J. T. Bouwman F. (2016). MRI visual rating scales in the diagnosis of dementia: evaluation in 184 post-mortem confirmed cases. *Brain* 139 1211–1225. 10.1093/brain/aww005 26936938 PMC4806219

[B42] Hodges J. R. Davies R. R. Xuereb J. H. Casey B. Broe M. Bak T. H. (2004). Clinicopathological correlates in frontotemporal dementia. *Ann. Neurol.* 56 399–406. 10.1002/ana.20203 15349867

[B43] Hornberger M. Piguet O. (2012). Episodic memory in frontotemporal dementia: a critical review. *Brain* 135 678–692. 10.1093/brain/aws011 22366790

[B44] Hornberger M. Piguet O. Graham A. J. Nestor P. J. Hodges J. R. (2010). How preserved is episodic memory in behavioral variant frontotemporal dementia? *Neurology* 74 472–479. 10.1212/WNL.0b013e3181cef85d 20142613 PMC2872800

[B45] Hu W. T. Chen-Plotkin A. Grossman M. Arnold S. E. Clark C. M. Shaw L. M. (2010). Novel CSF biomarkers for frontotemporal lobar degenerations. *Neurology* 75 2079–2086. 10.1212/WNL.0b013e318200d78d 21048198 PMC2995537

[B46] Illán-Gala I. Montal V. Borrego-Écija S. Vilaplana E. Pegueroles J. Alcolea D. (2019). Cortical microstructure in the behavioural variant of frontotemporal dementia: looking beyond atrophy. *Brain* 142 1121–1133. 10.1093/brain/awz031 30906945 PMC6439330

[B47] Irish M. Hornberger M. Lah S. Miller L. Pengas G. Nestor P. J. (2011). Profiles of recent autobiographical memory retrieval in semantic dementia, behavioural-variant frontotemporal dementia, and Alzheimer’s disease. *Neuropsychologia* 49 2694–2702. 10.1016/j.neuropsychologia.2011.05.017 21658396

[B48] Irish M. Piguet O. Hodges J. R. Hornberger M. (2014). Common and unique gray matter correlates of episodic memory dysfunction in frontotemporal dementia and Alzheimer’s disease. *Hum. Brain Mapp.* 35 1422–1435. 10.1002/hbm.22263 23670951 PMC6869668

[B49] Janelidze S. Hertze J. Zetterberg H. Landqvist Waldö M. Santillo A. Blennow K. (2016). Cerebrospinal fluid neurogranin and YKL-40 as biomarkers of Alzheimer’s disease. *Ann. Clin. Transl. Neurol.* 3 12–20. 10.1002/acn3.266 26783546 PMC4704480

[B50] Janssen J. C. Schott J. M. Cipolotti L. Fox N. C. Scahill R. I. Josephs K. A. (2005). Mapping the onset and progression of atrophy in familial frontotemporal lobar degeneration. *J. Neurol. Neurosurg. Psychiatry* 76 162–168. 10.1136/jnnp.2003.032201 15654025 PMC1739516

[B51] Jiskoot L. C. Bocchetta M. Nicholas J. M. Cash D. M. Thomas D. Modat M. (2018a). Presymptomatic white matter integrity loss in familial frontotemporal dementia in the GENFI cohort: a cross-sectional diffusion tensor imaging study. *Ann. Clin. Transl. Neurol.* 5 1025–1036. 10.1002/acn3.601 30250860 PMC6144447

[B52] Jiskoot L. C. Dopper E. G. P. Heijer T. den Timman R. van Minkelen R. van Swieten J. C. (2016). Presymptomatic cognitive decline in familial frontotemporal dementia: a longitudinal study. *Neurology* 87 384–391. 10.1212/WNL.0000000000002895 27358337

[B53] Jiskoot L. C. Panman J. L. Meeter L. H. Dopper E. G. P. Donker Kaat L. Franzen S. (2019). Longitudinal multimodal MRI as prognostic and diagnostic biomarker in presymptomatic familial frontotemporal dementia. *Brain* 142 193–208. 10.1093/brain/awy288 30508042 PMC6308313

[B54] Jiskoot L. C. Panman J. L. van Asseldonk L. Franzen S. Meeter L. H. H. Donker Kaat L. (2018b). Longitudinal cognitive biomarkers predicting symptom onset in presymptomatic frontotemporal dementia. *J. Neurol.* 265 1381–1392. 10.1007/s00415-018-8850-7 29627938 PMC5990575

[B55] Johnson J. K. Diehl J. Mendez M. F. Neuhaus J. Shapira J. S. Forman M. (2005). Frontotemporal lobar degeneration. *Arch. Neurol.* 62 925–930. 10.1001/archneur.62.6.925 15956163

[B56] Kertesz A. Ang L. C. Jesso S. MacKinley J. Baker M. Brown P. (2013). Psychosis and hallucinations in frontotemporal dementia with the C9ORF72 mutation: a detailed clinical cohort. *Cogn. Behav. Neurol.* 26 146–154. 10.1097/WNN.0000000000000008 24077574 PMC4090685

[B57] Krämer J. Lueg G. Schiffler P. Vrachimis A. Weckesser M. Wenning C. (2018). Diagnostic value of diffusion tensor imaging and positron emission tomography in early stages of frontotemporal dementia. *J. Alzheimers Dis.* 63 239–253. 10.3233/JAD-170224 29614640

[B58] Kuiperij H. B. Versleijen A. A. M. Beenes M. Verwey N. A. Benussi L. Paterlini A. (2017). Tau rather than TDP-43 proteins are potential cerebrospinal fluid biomarkers for frontotemporal lobar degeneration subtypes: a pilot study. *J. Alzheimer’s Dis.* 55 19–35. 10.3233/JAD-160386 27662293

[B59] Kämälaïnen A. Herukka S. K. Hartikainen P. Helisalmi S. Moilanen V. Knuuttila A. (2015). Cerebrospinal fluid biomarkers for Alzheimer’s disease in patients with frontotemporal lobar degeneration and amyotrophic lateral sclerosis with the C9ORF72 repeat expansion. *Dement. Geriatr. Cogn. Disord.* 39 287–293. 10.1159/000371704 25791939

[B60] Landqvist Waldö M. Gustafson L. Passant U. Englund E. (2015). Psychotic symptoms in frontotemporal dementia: a diagnostic dilemma? *Int. Psychogeriatr.* 27 531–539. 10.1017/S1041610214002580 25486967 PMC4413855

[B61] Lee S. E. Khazenzon A. M. Trujillo A. J. Guo C. C. Yokoyama J. S. Sha S. J. (2014). Altered network connectivity in frontotemporal dementia with C9orf72 hexanucleotide repeat expansion. *Brain* 137 3047–3060. 10.1093/brain/awu248 25273996 PMC4208465

[B62] Lee S. E. Sias A. C. Mandelli M. L. Brown J. A. Brown A. B. Khazenzon A. M. (2017). Network degeneration and dysfunction in presymptomatic C9ORF72 expansion carriers. *NeuroImage Clin.* 14 286–297. 10.1016/j.nicl.2016.12.006 28337409 PMC5349617

[B63] Lehmer C. Oeckl P. Weishaupt J. H. Volk A. E. Diehl-Schmid J. Schroeter M. L. (2017). Poly-GP in cerebrospinal fluid links C9orf72 -associated dipeptide repeat expression to the asymptomatic phase of ALS/FTD. *EMBO Mol. Med.* 9 859–868. 10.15252/emmm.201607486 28408402 PMC5494528

[B64] Liljegren M. Naasan G. Temlett J. Perry D. C. Rankin K. P. Merrilees J. (2015). Criminal behavior in frontotemporal dementia and Alzheimer disease. *JAMA Neurol.* 72 295–300. 10.1001/jamaneurol.2014.3781 25559744 PMC4432918

[B65] Lillo P. Garcin B. Hornberger M. Bak T. H. Hodges J. R. (2010). Neurobehavioral features in frontotemporal dementia with amyotrophic lateral sclerosis. *Arch. Neurol.* 67 826–830. 10.1001/archneurol.2010.146 20625088

[B66] Ljubenkov P. A. Staffaroni A. M. Rojas J. C. Allen I. E. Wang P. Heuer H. (2018). Cerebrospinal fluid biomarkers predict frontotemporal dementia trajectory. *Ann. Clin. Transl. Neurol.* 5 1250–1263. 10.1002/acn3.643 30349860 PMC6186942

[B67] Mahoney C. J. Beck J. Rohrer J. D. Lashley T. Mok K. Shakespeare T. (2012). Frontotemporal dementia with the C9ORF72 hexanucleotide repeat expansion: clinical, neuroanatomical and neuropathological features. *Brain* 135 736–750. 10.1093/brain/awr361 22366791 PMC3286330

[B68] Meeter L. H. Dopper E. G. Jiskoot L. C. Sanchez-Valle R. Graff C. Benussi L. (2016). Neurofilament light chain: a biomarker for genetic frontotemporal dementia. *Ann. Clin. Transl. Neurol.* 3 623–636. 10.1002/acn3.325 27606344 PMC4999594

[B69] Meeter L. H. H. Patzke H. Loewen G. Dopper E. G. P. Pijnenburg Y. A. L. van Minkelen R. (2016). Progranulin levels in plasma and cerebrospinal fluid in granulin mutation carriers. *Dement. Geriatr. Cogn. Dis. Extra.* 6 330–340. 10.1159/000447738 27703466 PMC5040889

[B70] Meeter L. H. H. Vijverberg E. G. Campo M. Del Rozemuller A. J. M. Kaat L. D. de Jong F. J. (2018). Clinical value of neurofilament and phospho-tau/tau ratio in the frontotemporal dementia spectrum. *Neurology* 90 e1231–e1239. 10.1212/wnl.0000000000005261 29514947 PMC5890612

[B71] Mendez M. F. Perryman K. M. (2002). Neuropsychiatric features of frontotemporal dementia: evaluation of consensus criteria and review. *J. Neuropsychiatry Clin. Neurosci.* 14 424–429. 10.1176/jnp.14.4.424 12426410

[B72] Menon V. Uddin L. Q. (2010). Saliency, switching, attention and control: a network model of insula function. *Brain Struct. Funct.* 214 655–667. 10.1007/s00429-010-0262-0 20512370 PMC2899886

[B73] Morbelli S. Ferrara M. Fiz F. Dessi B. Arnaldi D. Picco A. (2016). Mapping brain morphological and functional conversion patterns in predementia late-onset bvFTD. *Eur. J. Nucl. Med. Mol. Imaging* 43 1337–1347. 10.1007/s00259-016-3335-3 26928581

[B74] Moretti D. V. Benussi L. Fostinelli S. Ciani M. Binetti G. Ghidoni R. (2016). Progranulin mutations affects brain oscillatory activity in fronto-temporal dementia. *Front. Aging Neurosci.* 8:35. 10.3389/fnagi.2016.00035 26973510 PMC4770190

[B75] Niikado M. Chrem-Méndez P. Itzcovich T. Barbieri-Kennedy M. Calandri I. Martinetto H. (2018). Evaluation of cerebrospinal fluid neurofilament light chain as a routine biomarker in a memory clinic. *J. Gerontol. Ser. A* 74 442–445. 10.1093/gerona/gly179 30107413

[B76] Olm C. A. McMillan C. T. Irwin D. J. Van Deerlin V. M. Cook P. A. Gee J. C. (2018). Longitudinal structural gray matter and white matter MRI changes in presymptomatic progranulin mutation carriers. *NeuroImage Clin.* 19 497–506. 10.1016/j.nicl.2018.05.017 29984158 PMC6029561

[B77] Omar R. Sampson E. L. Loy C. T. Mummery C. J. Fox N. C. Rossor M. N. (2009). Delusions in frontotemporal lobar degeneration. *J. Neurol.* 256 600–607. 10.1007/s00415-009-0128-7 19365594 PMC2756566

[B78] Papma J. M. Jiskoot L. C. Panman J. L. Dopper E. G. den Heijer T. Donker Kaat L. (2017). Cognition and gray and white matter characteristics of presymptomatic C9orf72 repeat expansion. *Neurology* 89 1256–1264. 10.1212/WNL.0000000000004393 28855404

[B79] Pardini M. Emberti Gialloreti L. Mascolo M. Benassi F. Abate L. Guida S. (2013). Isolated theory of mind deficits and risk for frontotemporal dementia: a longitudinal pilot study. *J. Neurol. Neurosurg. Psychiatry* 84 818–821. 10.1136/jnnp-2012-303684 23117487

[B80] Paterson R. W. Slattery C. F. Poole T. Nicholas J. M. Magdalinou N. K. Toombs J. (2018). Cerebrospinal fluid in the differential diagnosis of Alzheimer’s disease: clinical utility of an extended panel of biomarkers in a specialist cognitive clinic. *Alzheimers Res. Ther.* 10:32 10.1186/s13195-018-0361-3 PMC5861624 29558979

[B81] Pennington C. Hodges J. R. Hornberger M. (2011). Neural correlates of episodic memory in behavioral variant frontotemporal dementia. *J. Alzheimer’s Dis.* 24 261–268. 10.3233/JAD-2011-101668 21239854

[B82] Perry D. C. Brown J. A. Possin K. L. Datta S. Trujillo A. Radke A. (2017). Clinicopathological correlations in behavioural variant frontotemporal dementia. *Brain* 140 3329–3345. 10.1093/brain/awx254 29053860 PMC5841140

[B83] Pievani M. de Haan W. Wu T. Seeley W. W. Frisoni G. B. (2011). Functional network disruption in the degenerative dementias. *Lancet Neurol.* 10 829–843. 10.1016/S1474-4422(11)70158-2 21778116 PMC3219874

[B84] Pievani M. Paternicò D. Benussi L. Binetti G. Orlandini A. Cobelli M. (2014). Pattern of structural and functional brain abnormalities in asymptomatic granulin mutation carriers. *Alzheimers Dement.* 10 S354–S363. 10.1016/j.jalz.2013.09.009 24418059

[B85] Pijnenburg Y. A. L. Gillissen F. Jonker C. Scheltens P. (2004). Initial complaints in frontotemporal lobar degeneration. *Dement. Geriatr. Cogn. Disord.* 17 302–306. 10.1159/000077159 15178941

[B86] Pijnenburg Y. A. L. Strijers R. L. M. Made Y. V. van der Flier W. M. Scheltens P. Stam C. J. (2008). Investigation of resting-state EEG functional connectivity in frontotemporal lobar degeneration. *Clin. Neurophysiol.* 119 1732–1738. 10.1016/j.clinph.2008.02.024 18490193

[B87] Pijnenburg Y. A. L. Verwey N. A. van der Flier W. M. Scheltens P. Teunissen C. E. (2015). Discriminative and prognostic potential of cerebrospinal fluid phosphoTau/tau ratio and neurofilaments for frontotemporal dementia subtypes. *Alzheimer’s Dement. Diagn. Assess. Dis. Monit.* 1 505–512. 10.1016/j.dadm.2015.11.001 27239528 PMC4879490

[B88] Poletti M. Enrici I. Adenzato M. (2012). Cognitive and affective Theory of Mind in neurodegenerative diseases: neuropsychological, neuroanatomical and neurochemical levels. *Neurosci. Biobehav. Rev.* 36 2147–2164. 10.1016/j.neubiorev.2012.07.004 22819986

[B89] Popuri K. Dowds E. Beg M. F. Balachandar R. Bhalla M. Jacova C. (2018). Gray matter changes in asymptomatic C9orf72 and GRN mutation carriers. *NeuroImage Clin.* 18 591–598. 10.1016/j.nicl.2018.02.017 29845007 PMC5964622

[B90] Ranasinghe K. G. Rankin K. P. Lobach I. V. Kramer J. H. Sturm V. E. Bettcher B. M. (2016). Cognition and neuropsychiatry in behavioral variant frontotemporal dementia by disease stage. *Neurology* 86 600–610. 10.1212/WNL.0000000000002373 26802093 PMC4762418

[B91] Rascovsky K. Hodges J. R. Knopman D. Mendez M. F. Kramer J. H. Neuhaus J. (2011). Sensitivity of revised diagnostic criteria for the behavioural variant of frontotemporal dementia. *Brain* 134 2456–2477. 10.1093/brain/awr179 21810890 PMC3170532

[B92] Ratnavalli E. Brayne C. Dawson K. Hodges J. R. (2002). The prevalence of frontotemporal dementia. *Neurology* 58 1615–1621. 12058088 10.1212/wnl.58.11.1615

[B93] Riemenschneider M. Wagenpfeil S. Vanderstichele H. Otto M. Wiltfang J. Kretzschmar H. (2003). Phospho-tau/total tau ratio in cerebrospinal fluid discriminates creutzfeldt-Jakob disease from other dementias. *Mol. Psychiatry* 8 343–347. 10.1038/sj.mp.4001220 12660807

[B94] Rivero-Santana A. Ferreira D. Perestelo-Pérez L. Westman E. Wahlund L. O. Sarría A. (2017). Cerebrospinal fluid biomarkers for the differential diagnosis between alzheimer’s disease and frontotemporal lobar degeneration: systematic review, HSROC analysis, and confounding factors. *J. Alzheimer’s Dis.* 55 625–644. 10.3233/JAD-160366 27716663

[B95] Rohrer J. D. Nicholas J. M. Cash D. M. van Swieten J. Dopper E. Jiskoot L. (2015). Presymptomatic cognitive and neuroanatomical changes in genetic frontotemporal dementia in the genetic frontotemporal dementia initiative (GENFI) study: a cross-sectional analysis. *Lancet Neurol.* 14 253–262. 10.1016/S1474-4422(14)70324-2 25662776 PMC6742501

[B96] Rohrer J. D. Warren J. D. Barnes J. Mead S. Beck J. Pepple T. (2008). Mapping the progression of progranulin-associated frontotemporal lobar degeneration. *Nat. Clin. Pract. Neurol.* 4 455–460. 10.1038/ncpneuro0869 18648346 PMC2567307

[B97] Rosness T. A. Haugen P. K. Passant U. Engedal K. (2008). Frontotemporal dementia: a clinically complex diagnosis. *Int. J. Geriatr. Psychiatry* 23 837–842. 10.1002/gps.1992 18302319

[B98] Rytty R. Nikkinen J. Paavola L. Abou Elseoud A. Moilanen V. Visuri A. (2013). GroupICA dual regression analysis of resting state networks in a behavioral variant of frontotemporal dementia. *Front. Hum. Neurosci.* 7:461. 10.3389/fnhum.2013.00461 23986673 PMC3752460

[B99] Seeley W. W. Crawford R. K. Zhou J. Miller B. L. Greicius M. D. (2009). Neurodegenerative diseases target large-scale human brain networks. *Neuron* 62 42–52. 10.1016/j.neuron.2009.03.024 19376066 PMC2691647

[B100] Shinagawa S. Shigenobu K. Tagai K. Fukuhara R. Kamimura N. Mori T. (2017). Violation of laws in frontotemporal dementia: a multicenter study in Japan. *J. Alzheimers Dis.* 57 1221–1227. 10.3233/JAD-170028 28304308

[B101] Skillbäck T. Farahmand B. Bartlett J. W. Rosén C. Mattsson N. Nägga K. (2014). CSF neurofilament light differs in neurodegenerative diseases and predicts severity and survival. *Neurology* 83 1945–1953. 10.1212/WNL.0000000000001015 25339208

[B102] Snowden J. S. Rollinson S. Thompson J. C. Harris J. M. Stopford C. L. Richardson A. M. T. (2012). Distinct clinical and pathological characteristics of frontotemporal dementia associated with C9ORF72 mutations. *Brain* 135 693–708. 10.1093/brain/awr355 22300873 PMC3286329

[B103] Solje E. Aaltokallio H. Koivumaa-Honkanen H. Suhonen N. M. Moilanen V. Kiviharju A. (2015). The phenotype of the C9ORF72 expansion carriers according to revised criteria for bvFTD. *PLoS One* 10:E131817. 10.1371/journal.pone.0131817 26146826 PMC4493025

[B104] Steinacker P. Anderl-Straub S. Diehl-Schmid J. Semler E. Uttner I. von Arnim C. A. F. (2018). Serum neurofilament light chain in behavioral variant frontotemporal dementia. *Neurology* 91 e1390–e1401. 10.1212/wnl.0000000000006318 30209235

[B105] Su Z. Zhang Y. Gendron T. F. Bauer P. O. Chew J. Yang W. Y. (2014). Discovery of a biomarker and lead small molecules to target r(GGGGCC)-associated defects in c9FTD/ALS. *Neuron* 83 1043–1050. 10.1016/j.neuron.2014.07.041 25132468 PMC4232217

[B106] Velakoulis D. Walterfang M. Mocellin R. Pantelis C. McLean C. (2009). Frontotemporal dementia presenting as schizophrenia-like psychosis in young people: clinicopathological series and review of cases. *Br. J. Psychiatry* 194 298–305. 10.1192/bjp.bp.108.057034 19336778

[B107] Vijverberg E. G. B. Dols A. Krudop W. A. Milan M. D. C. Kerssens C. J. Gossink F. (2017). Cerebrospinal fluid biomarker examination as a tool to discriminate behavioral variant frontotemporal dementia from primary psychiatric∼disorders. *Alzheimers Dement. Diagn. Assess. Dis. Monit.* 7 99–106. 10.1016/j.dadm.2017.01.009 28337476 PMC5352718

[B108] Walhout R. Schmidt R. Westeneng H.-J. Verstraete E. Seelen M. van Rheenen W. (2015). Brain morphologic changes in asymptomatic C9orf72 repeat expansion carriers. *Neurology* 85 1780–1788. 10.1212/WNL.0000000000002135 26497991

[B109] Wen J. Zhang H. Alexander D. C. Durrleman S. Routier A. Rinaldi D. (2018). Neurite density is reduced in the presymptomatic phase of C9orf72 disease. *J. Neurol. Neurosurg. Psychiatry* 90 387–394. 10.1136/jnnp-2018-318994 30355607

[B110] Whitwell J. L. Josephs K. A. Avula R. Tosakulwong N. Weigand S. D. Senjem M. L. (2011). Altered functional connectivity in asymptomatic MAPT subjects a comparison to bvFTD. *Neurology* 77 866–874. 10.1212/WNL.0b013e31822c61f2 21849646 PMC3162637

[B111] Woollacott I. O. C. Nicholas J. M. Heslegrave A. Heller C. Foiani M. S. Dick K. M. (2018). Cerebrospinal fluid soluble TREM2 levels in frontotemporal dementia differ by genetic and pathological subgroup. *Alzheimers Res. Ther.* 10:79 10.1186/s13195-018-0405-8 PMC6094471 30111356

[B112] Zhou J. Greicius M. D. Gennatas E. D. Growdon M. E. Jang J. Y. Rabinovici G. D. (2010). Divergent network connectivity changes in behavioural variant frontotemporal dementia and Alzheimer’s disease. *Brain* 133 1352–1367. 10.1093/brain/awq075 20410145 PMC2912696

